# Pheochromocytoma as a rare cause of hypertension in a 46 X, i(X)(q10) turner syndrome: a case report and literature review

**DOI:** 10.1186/s12902-018-0253-3

**Published:** 2018-05-10

**Authors:** Ji Yeon Shin, Bo Hyun Kim, Young Keum Kim, Tae Hwa Kim, Eun Heui Kim, Min Jin Lee, Jong Ho Kim, Yun Kyung Jeon, Sang Soo Kim, In Joo Kim

**Affiliations:** 1 Department of Internal Medicine, Pusan National University College of Medicine, Busan, 49241 South Korea; 2 Biomedical Research Institute, Pusan National University Hospital, Busan, 49241 South Korea; 3 Department of Pathology, Pusan National University Hospital and Pusan National University School of Medicine, Busan, 49241 South Korea; 4 Division of Endocrinology and Metabolism, Department of Internal Medicine, Pusan National University Hospital, 305 Gudeok-ro, Seo-gu, Busan, 602-739 South Korea

**Keywords:** Hypertension, Turner syndrome, Pheochromocytoma

## Abstract

**Background:**

Cardiovascular disease (CVD) presents the most serious health problems and contributes to the increased mortality in young women with Turner syndrome. Arterial hypertension in Turner syndrome patients is significantly more prevalent than that in a general age-matched control group. The aetiology of hypertension in Turner syndrome varies, even in the absence of cardiac anomalies and obvious structural renal abnormalities. Pheochromocytoma is an extremely rare cause among various etiologies for hypertension in patients with Turner syndrome. Here, we reported a pheochromocytoma as a rare cause of hypertension in Turner syndrome patient.

**Case presentation:**

A 21-year-old woman who has diagnosed with Turner syndrome with a karyotype of 46,X,i(X)(q10) visited for hypertension and mild headache. Transthoracic echography (TTE) showed no definite persistent ductus arteriosus shunt flow and cardiac valve abnormalities. Considering other important secondary causes like pheochromocytoma, hormonal studies were performed and the results showed increased serum norepinephrine, serum normetanephrine, and 24 h urine norepinephrine. We performed an abdominal computed tomography (CT) to confirm the location of pheochromocytoma. Abdominal CT showed a 1.9 cm right adrenal mass. I-131 meta-iodobenzylguanidine (MIBG) scintigraphy showed a right adrenal uptake. Laparoscopic adrenalectomy was performed and confirmed a pheochromocytoma. After surgery, blood pressure was within normal ranges and postoperative course was uneventful, and no recurrence developed via biochemical tests and abdominal CT until 24 months.

**Conclusion:**

Our case and previous literatures suggest that hypertension caused by pheochromocytoma which is a rare but important and potentially lethal cause of hypertension in Turner syndrome. This case underlines the importance of early detection of pheochromocytoma in Turner syndrome. Clinicians should keep in mind that pheochromocytoma can be a cause of hypertension in patients with Turner syndrome.

## Background

Turner syndrome is well known as a common chromosomal disorder with complete or partial absence of one X chromosome. Monosomy X is present in approximately 50% of cases. It occurs only in women between approximately 1/2500 and 1/5000 live female births. Turner syndrome represents an important cause of short stature and ovarian insufficiency in females [1]. In addition to these typical phenotypes, congenital and acquired cardiovascular disease (CVD), diabetes, hypothyroidism, impaired hearing, scoliosis, renal abnormalities and neurocognitive disorders are frequently associated with Turner syndrome [1, 2]. Among these diseases, CVDs such as cardiovascular malformation and coronary artery disease present the most serious health problems and contribute to the increased mortality rates. Importantly, arterial hypertension occurs frequently in Turner syndrome patients with an estimated prevalence of 13 to 58% in girls. Hypertension significantly increases the risk for CVD and aortic dissection [3–6]. Congenital malformation such as coarctation of the aorta and renal abnormalities, which can cause hypertension, can often be seen in patients. In addition, aortic dissection is a fatal complication in patients with Turner syndrome and often occurs at a young age [7]. Although the cause of hypertension in Turner syndrome is still not clear, structural etiology as well as heart autonomic innervations, increased plasma renin activity (PRA), and parasympathetic neuropathy, can cause hypertension in Turner syndrome patients. Thus, hypertension is the most important modifiable risk factor for aortic dissection and rupture that occurs in young women with Turner syndrome [3, 8–10]. Therefore, adults with Turner syndrome should undergo regular cardiac examinations and imaging studies to detect aortic root dilatation, cardiac valve disease, or other cardiovascular anomalies, as well as routine blood pressure (BP) monitoring [4]. However, according to the English literature, pheochromocytoma is an extremely rare cause among various aetiologies for hypertension in patients with Turner syndrome [11–13]. Here, we present a rare case of a female patient with 46 X, i(X)(q10) Turner syndrome and hypertension due to pheochromocytoma and review previous cases according to the literatures.

## Case presentation

A 21-year-old woman was referred due to secondary amenorrhea. Her past medical history was significant for persistent ductus arteriosus (PDA), she underwent surgery when she was 11 months old, and she was diagnosed with type 2 diabetes mellitus (DM), which was incidentally detected at the age of 18 years. At that time, the physical examination findings were as follows: height of 155 cm, body weight of 80 kg, body mass index (BMI) of 33.3 kg/m^2^, and BP of 108/76 mmHg. After being diagnosed with type 2 DM, the patient took metformin for a few months, and she tried to lose weight through exercise and diet control. Recently, although she had not taken metformin for several months, her blood glucose was within normal ranges. At this visit, a physical examination showed a height of 155.3 cm and a weight of 53.7 kg, with a BMI of 22.3 kg/m^2^. Her BP was 130/80 mmHg, with a regular heart rate of 80 beats/min, and her body temperature was 36.5 °C. The development of her breasts and her pubic hair were Tanner stage 2 and stage 3, respectively. The female internal genitalia were infantile. However, there was no webbed neck or skeletal deformities.

**Fig. 1:**
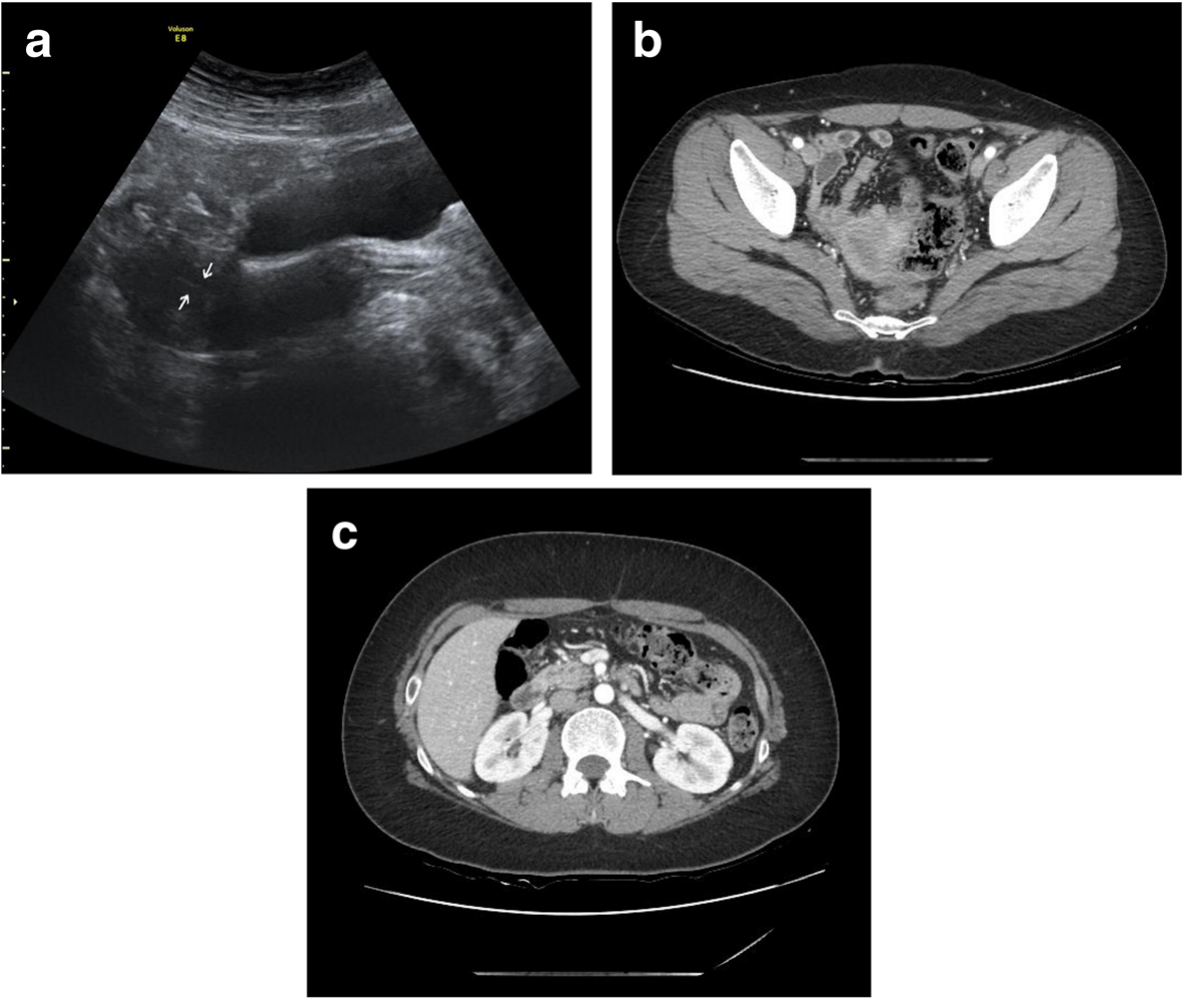
Ultrasonography image (**a**) and abdomen computed tomography axial image with contrast enhancement (**b**) shows small uterus and both ovaries are not visible. There was no abnormalities of urogenital system (**c**)

Laboratory tests revealed a fasting glucose level of 88 mg/dL, HbA1C 4.8%, C-peptide 6.34 ng /mL(reference range, 0.4–4 ng/mL), and anti-glutamate decarboxylase (GAD) antibody < 0.5 U/mL(reference range, 0–1.0 U/mL). Hormonal tests showed a thyroid-stimulating hormone (TSH) level of 2.45 μIU/mL (reference range, 0.3–5.0 μIU/mL), a T3 level of 169.8 ng/dL (reference range, 80–200 ng/dL), a free T4 level of 1.15 ng/dL (reference range, 0.75–2.0 ng/dL), a prolactin level of 8.18 ng/mL (reference range, 1.9–19.7 ng/mL), a luteinizing hormone (LH) level of 20.2 mIU/mL, a follicular stimulating hormone (FSH) level of 100 mIU/mL, a testosterone level of 0.01 ng/mL (reference range, 0–0.77 ng/mL), an oestradiol level of 1.06 μg/dL(reference range, 30–333 μg/dL). A small uterus was detected, and both ovaries could not be identified on pelvis ultrasonography and computed tomography (CT) (Fig. 1a, b). However, there was no renal abnormality (Fig. 1c). A chromosome study finally showed a karyotype of 46,X,i (X)(q10) Turner syndrome. Transthoracic echography (TTE) revealed no residual PDA shunt flow and bicuspid valve. Bone mineral density (BMD) using dual-energy X-ray absorptiometry showed an age-matched Z score of − 0.7 at the lumbar spine. She was regularly followed up for type 2 DM and took ethinyl oestradiol/drospirenone for regular menstruation.

**Fig. 2:**
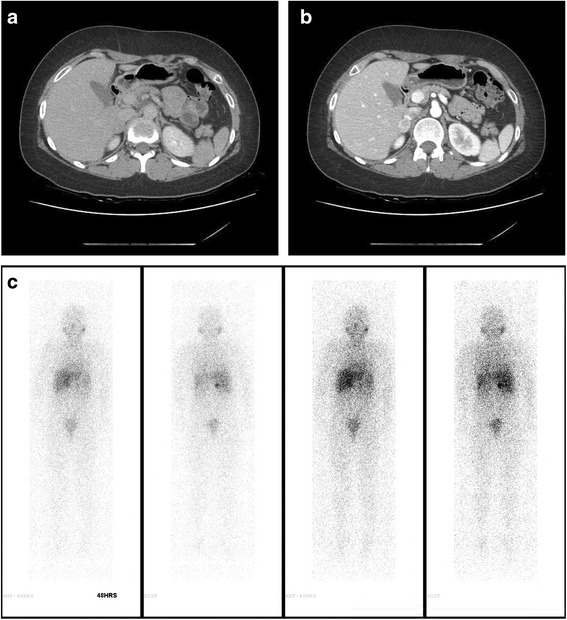
(**a**) Abdomen computed tomography axial image with contrast enhancement shows a well-defined mass measuring 2 cm in right adrenal gland. Hounsfield unit (HU) at pre-contrast enhancement image is 42 HU, 138 HU at 1 min delayed image. (**b**) 10 min delayed image show a 75 HU and washout ratio is 65%. (**c**) I-131 metaiodobenzylguanidine (MIBG) image show a mass with increased uptake in right adrenal gland

**Fig. 3:**
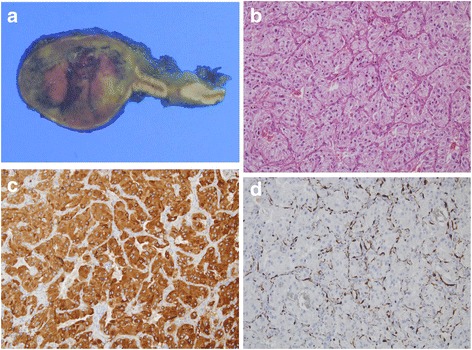
The cut surface of the tumour was tan and darkened, with focal haemorrhage and extended to the adrenal cortex but was well-circumscribed (**a**). The tumour showed characteristic “Zellballen” architecture and tumor cells were larger than normal chromaffin cells, and their cytoplasm was granular (**b**). Immunostaining for S-100 protein demonstrate the sustentacular framework surrounding the tumor cells (**c**) and positive for chromogranin (**d**)

Three years after the diagnosis of Turner syndrome, the patient complained of mild headache, intermittent palpitation, and dizziness without neurological deficit. Her measured BP was 166/100 mmHg at the doctor’s office and 145/90 mmHg at home. Her BP at the time of the Turner syndrome diagnosis (3 years ago) was within normal ranges. There was no radio-femoral delay which meant delay between the radial pulse and femoral pulse suggesting coarctation of aorta. Recent TTE showed no residual PDA shunt flow and cardiac valve abnormality. There was no family history of hypertension. A thyroid function test and chemistry were within normal ranges. We consider other important secondary causes of hypertension like a pheochromocytoma. Thus, hormonal studies were performed and the results showed increased serum norepinephrine, serum normetanephrine, and 24 h urine norepinephrine (Table 1). We performed an abdominal computed tomography (CT) to confirm the location of pheochromocytoma. Abdominal CT showed a 1.9 cm right adrenal mass, measuring 42 Hounsfield units pre-contrast, heterogenous contrast enhancement with a contrast wash-out ratio of 65% (Fig. 2a and b). According to her clinical manifestations, biochemical results and atypical CT phenotype, I-131 meta-iodo-benzylguanidine (MIBG) scintigraphy was performed to detect of extra-adrenal pheochromocytoma. The results showed MIBG uptake on the right adrenal gland (Fig. 2c). She had no family history of pheochromocytoma. After pre-operatively administering phenoxybenzamine, laparoscopic adrenalectomy was performed. Final pathology revealed that the pheochromocytoma extended to the adrenal cortex but was well-circumscribed. The cut surface of the tumour was tan and darkened with focal haemorrhage. The tumour showed characteristic “Zellballen” architecture. The tumour cells were larger than normal chromaffin cells, and their cytoplasm was granular. Immunostaining for S-100 protein demonstrates the sustentacular framework surrounding the tumour cells and positive for chromogranin (Fig. 3). Postoperative blood pressure was recorded as 120/70 mmHg without any anti-hypertensive drugs. Postoperative hormonal tests revealed decreased catecholamine levels (Table 1). The postoperative course was uneventful, and no recurrence developed via biochemical tests and abdominal CT until 24 months.

**Table 1:** Laboratory findings for adrenal gland mass on admission and post-operation

Parameters	Pre-operative value	Post-operative value	Reference ranges
Serum norepinephrine, pg/mL	1066.2	294.0	110–410
Serum epinephrine, pg/mL	17.5	14.4	< 50
Serum normetanephrine, nmol/L	3.32	0.5	< 0.9
Serum metanephrine, nmol/L	0.13	0.12	< 0.5
Urinary norepinephrine, μg/24hours^a^	282.6	34.4	0–97
Urinary epinephrine, μg/24hours^a^	4.35	3.6	0–27
Urinary normetanephrine, μg/24hours^a^	399.9	176.3	88–444
Urinary metanephrine, μg/24hours^a^	21.3	58.6	52-341
Urinary VMA, mg/day	4.72	2.04	1.2–6.52
Serum ACTH, pg/mL	10.38	16.15	10–60
Serum cortisol, μg/dL	45	40.0	2.5–12.5
24 h urine free cortisol, μg/24 hours^a^	49.7	45.4	7–96
Plasma renin activity, ng/ml/hr	9.14	5.16	0.15–2.33
Serum aldosterone, pg/mL	864.4	199.7	10–160
Aldosterone renin ratio	9.46	3.9	< 30
Serum Potassium, mmol/L	4.23	3.92	3.5–5.3

^a^24 hours urine total volume 1650 mL, urinary creatinine 1171.9 mg/24 h

## Discussion

In this article, we reported a rare case of hypertension caused by pheochromocytoma in a young woman with 46 X, i(X)(q10) Turner syndrome. Hypertension in Turner syndrome patients is significantly more prevalent than that in a general age-matched control group [4, 5, 10]. The aetiology of hypertension in Turner syndrome varies, even in the absence of cardiac anomalies and obvious structural renal abnormalities [3, 8–10]. Adults with Turner syndrome should undergo regular cardiac examinations and imaging studies to detect cardiovascular abnormalities, as well as routine check-ups of systemic BP, because CVD is the primary cause of mortality in Turner syndrome. Considering these points, hypertension is the most easily detectable and treatable cardiovascular risk factor to prevent CVD [4].

Turner syndrome patients usually are overweight; have central obesity, truncal fat mass, short stature, and high BMI; and are at risk for diabetes, which can cause cardiovascular abnormalities [6, 14, 15]. Turner syndrome is also associated with vascular wall abnormalities that are both structural and functional. Although some factors in the pathogenesis of hypertension remain uncertain, the potential pathogenesis includes inappropriate activation of the renin–angiotensin–aldosterone system, oxidative stress, inflammation, impaired insulin-mediated vasodilatation, increased stimulation of the sympathetic nervous system and abnormal sodium processing by the kidney [6, 16]. PRA has been found to be elevated in approximately 50% of Turner syndrome patients compared to the general population [8]. Over-activated autonomic innervations of the heart in Turner syndrome can increase the heart rate and BP [3]. Increased C-reactive protein is observed in half of Turner syndrome patients, which results in a chronic inflammatory state in the vascular endothelium and can likely lead to hypertension due to endothelial dysfunction [17]. In addition, hypertension with Turner syndrome is related to aortic stenosis, malformation of the urogenital system and other cardiac malformations [6]. High-dose growth hormone (GH) treatment causes hypertension due to sodium and water retention with increased levels of PRA and aldosterone levels [18]. However, in our case, the patient had no residual PDA shunt flow and cardiac valve abnormality and renal abnormalities and she did not receive GH treatment.

It is well-known that pheochromocytoma is one of the rare but important and potentially lethal causes of endocrine hypertension. Pheochromocytoma in patients with Turner syndrome is rarely reported, according to the English language literature [11–13]. Table 2 summarizes the clinical features and cardiovascular outcomes in previously reported cases. For the first time, Kinsely et al. reported an autopsy case with diagnosed pheochromocytoma and sudden death caused by haemorrhagic cerebral infarction in Turner syndrome [11]. Landin-Wilhelmsen et al. also reported a case of aortic dissection and pheochromocytoma in a case of Turner syndrome [12]. Recently, Fatma et al. reported that Turner syndrome with 45,X/46,X,i(Xq) karyotype was associated with incidentally detected pheochromocytoma [13]. In our case, the size of the pheochromocytoma was the smallest compared with previous cases. Unfortunately, the former two cases showed that pheochromocytoma in Turner syndrome led to poor CVD outcomes due to delayed diagnosis. Therefore, early detection and treatment of pheochromoctyoma is very important to prevent CVD and reduce mortality in young women with Turner syndrome. In case of clinical suspicion of pheochromocytoma, biochemical testing including plasma free or urinary metanephrines should be measured. In our case, serum norepinephrine, serum normetanephrine, and urinary norepinephrine were increased. However, urinary normetanephrine was within normal reference value. It is well known that the very high diagnostic sensitivity of metanephrines is due to the continuous diffusion of intratumorally-produced metanephrines into the circulation, which contrasts with the episodic secretion of the parent catecholamines [19]. However, it is not clear why the value of urinary normetanephrine was normal in this case. Previous two studies also reported increased metanephrine and normetanephrine [12, 13], however, levels of catecholamines and their catabolic products in serum or urine had not been determined in Knisely et al. study [11].

**Table 2:** The summary of the reported cases of pheochromocytoma in patient with Turner syndrome

Authors, year of publication (reference)	Age	Karyotype	Size of pheochromocytoma	Clinical manifestations	Cardiovascular outcomes
Knisely et al. (1988) [11]	27	45,XO	7.5 cm	Hemorrhagic cerebral infarct	Sudden death
Landin-Wilhelmsen et al. (2004) [12]	39	45,X[46]/46,X + mar [4]	3.0 cm	Chest pain hypertension	Aortic dissection
Fatma et al. (2016) [13]	48	46 X,i(Xq)/45X	6.0 cm	Adrenal incidentaloma hypertension	Curative hypertension
In our case	21	46 X, i(X)(q10)	1.9 cm	Hypertension	Curative hypertension

Although cancer risks in Turner syndrome except having an increased gonadoblastoma have not been clearly established, high incidence of extragonadal neoplasms with a preponderance of neurogenic tumors has been reported [20–22]. Although the overall risk of cancer was not increased in a population-based study, women with Turner syndrome had an increase of site-specific risk for gonadoblastoma, meningioma, childhood brain tumors, bladder, and uterine cancer when compared with the general population [23]. However, the clinical importance of this result was unclear due to very small number of cases. Like this population-based study, the related mechanisms between Turner syndrome and pheochromocytoma remain unclear, and it is also uncertain whether pheochromocytoma is over-represented in Turner syndrome because coexistence of these two diseases is extremely rare. Pheochromocytomas are known to be hereditary in 30–40% of cases, and hereditary catecholamine-secreting tumors typically present at a younger age than sporadic tumors [24]. Genetic testing should be considered in all patients and is strongly indicated in specific patients such as those with unilateral adrenal pheochromocytoma onset at a young age, those with a positive family history of pheochromocytoma and paraganglioma or carriers of tumor susceptibility gene mutations, and those with syndromic features or metastatic disease [19]. However, unfortunately, our patient had no family history of pheochromocytoma and she refused a genetic testing due to expensive cost of genetic testing. Further studies including genetic tests are necessary to define the mechanisms underlying association between Turner syndrome and pheochromcytoma.

## Conclusion

Our case and data from previous literature suggest that hypertension caused by pheochromocytoma which is a rare but important and potentially fatal cause of hypertension in Turner syndrome. This case underlines the importance of early detection of pheochromocytoma for prevention of CVD and reduction of mortality in young women with Turner syndrome. Clinicians should keep in mind that pheochromocytoma can be a cause of hypertension in patients with Turner syndrome. Thus, adults with Turner syndrome should undergo regular cardiac examinations, as well as routine BP monitoring. Furthermore, the possible association between Turner syndrome and pheochromocytoma should be elucidated in future studies.

## Abbreviations

BMD: Bone mineral density
BMI: Body mass index
BP: Blood pressure
CT: Computed tomography
CVD: Cardiovascular disease
DM: Diabetes mellitus
FSH: Follicular stimulating hormone
GAD: Glutamate decarboxylase
GH: Growth hormone
HU: Hounsfield unit
LH: Luteinizing Hormone
MIBG: Meta-iodobenzylguanidine
PDA: Persistent ductus arteriosus
PRA: Plasma renin activity
TSH: Thyroid-stimulating hormone
TTE: Transthoracic echography
